# Iatrogenic Arteriovenous Fistula of Subclavian Artery to Vertebral Vein with Perimedullary Vein Reflux

**DOI:** 10.3400/avd.cr.22-00016

**Published:** 2022-09-25

**Authors:** Koji Sato, Yasushige Shingu, Masato Fusegawa, Takahiro Ishigaki, Satoru Wakasa

**Affiliations:** 1 Department of Cardiovascular and Thoracic Surgery, Faculty of Medicine and Graduate School of Medicine, Hokkaido University, Sapporo, Hokkaido, Japan

**Keywords:** iatrogenic arteriovenous fistula, covered stent, subclavian artery

## Abstract

Iatrogenic arteriovenous fistula (AVF) rarely develops around the proximal subclavian artery, although open surgical repair of this etiology is known to be complicated as deep dissection is required around the fistula surrounded by dilated veins. In this study, we present the case of a 64-year-old man, who was referred to our hospital, with AVF between the right subclavian artery and the right vertebral vein. He had a history of accidental puncture of the right subclavian artery. An endovascular repair using a covered stent was successfully performed, and the AVF disappeared. Thus, covered stent placement should be considered as the first-line treatment for a deeply developed AVF, if anatomically feasible.

## Introduction

A mispuncture of the subclavian artery can occur during central venous catheter placement^1)^; this, in turn, can cause an arteriovenous fistula (AVF).^2)^ Open repair for an injury to the proximal part of the subclavian artery has been identified as a complicated and potentially invasive procedure because it requires deep dissection around the injury site that is surrounded by hematoma and tissues containing expanded and pressurized veins and could require a sternotomy or a thoracotomy. In contrast, an endovascular repair is safe and less invasive; however, this could be associated with a risk of occlusion of the branch arteries. Herein, we present a rare case of AVF between the subclavian artery and the vertebral vein, which was successfully treated with a covered stent.

## Case Report

**Figure 1:**
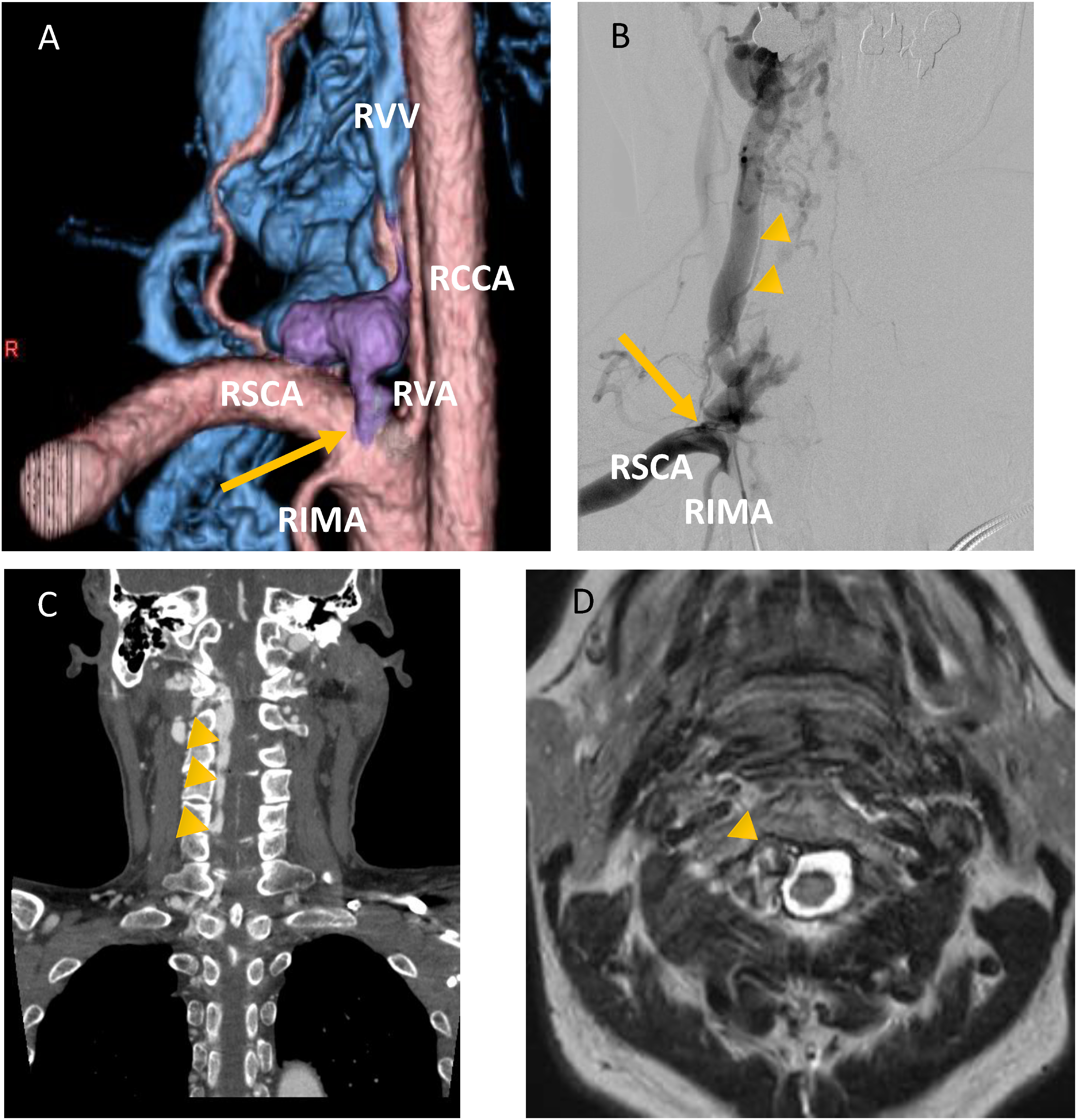
Fig. 1 Arteriovenous fistula and resultant epidural venous dilatation in preoperative computed tomography (**A** and **C**), angiography (**B**), and magnetic resonance imaging (**D**). The fistula was found between the right subclavian artery and the right vertebral vein (arrow, **A** and **B**). Epidural veins were dilated and compressed the spinal cord (arrowheads, **B**–**D**).

RCCA: right common carotid artery; RIMA: right internal mammary artery; RSCA: right subclavian artery; RVA: right vertebral artery; RVV: right vertebral vein

**Figure 2:**
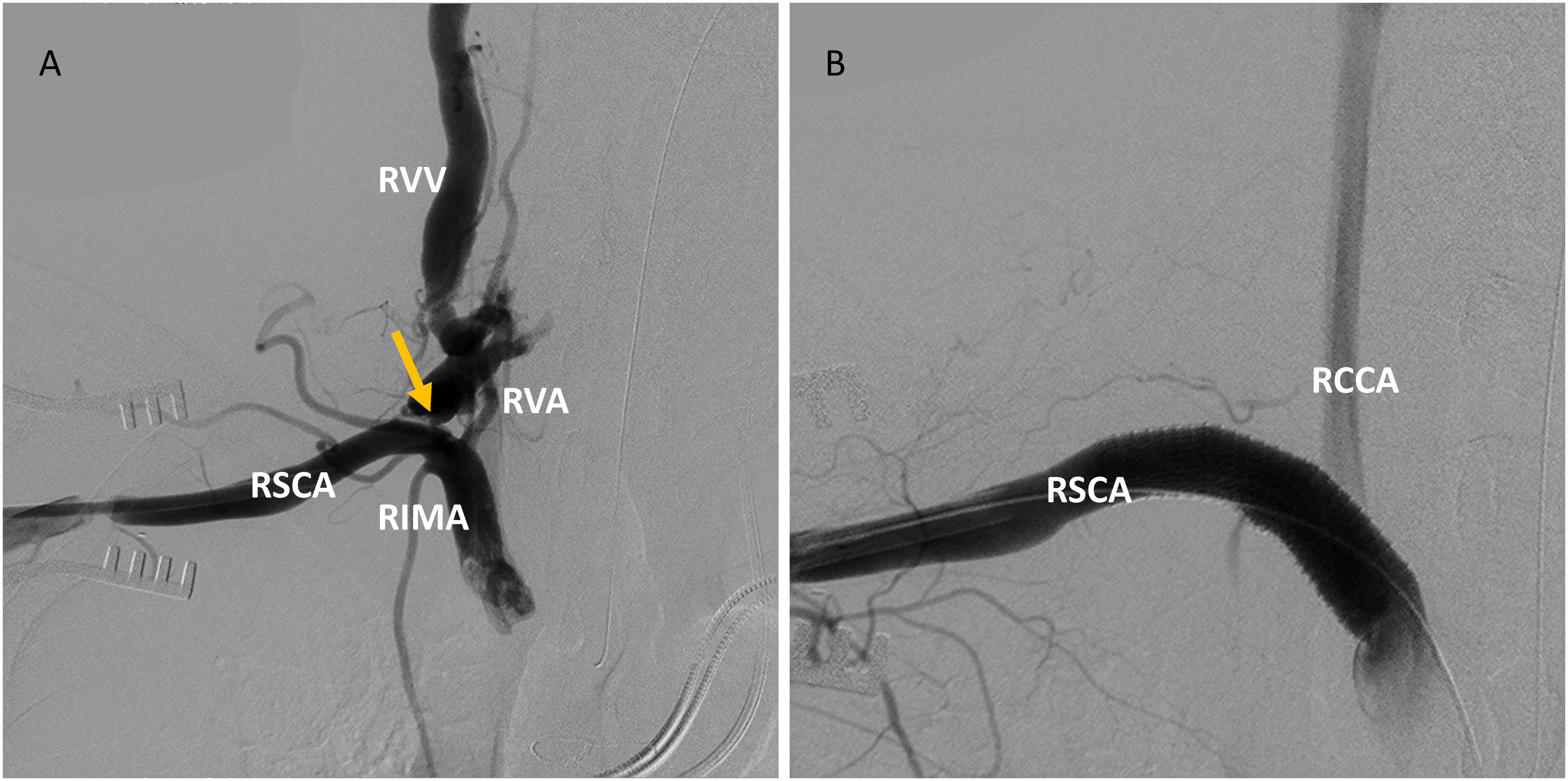
Fig. 2 Intraoperative digital subtraction angiography before (**A**) and after (**B**) deployment of covered stent. The arrow presented the site of fistula.

RCCA: right common carotid artery; RIMA: right internal mammary artery; RSCA: right subclavian artery; RVA: right vertebral artery; RVV: right vertebral vein

**Figure 3:**
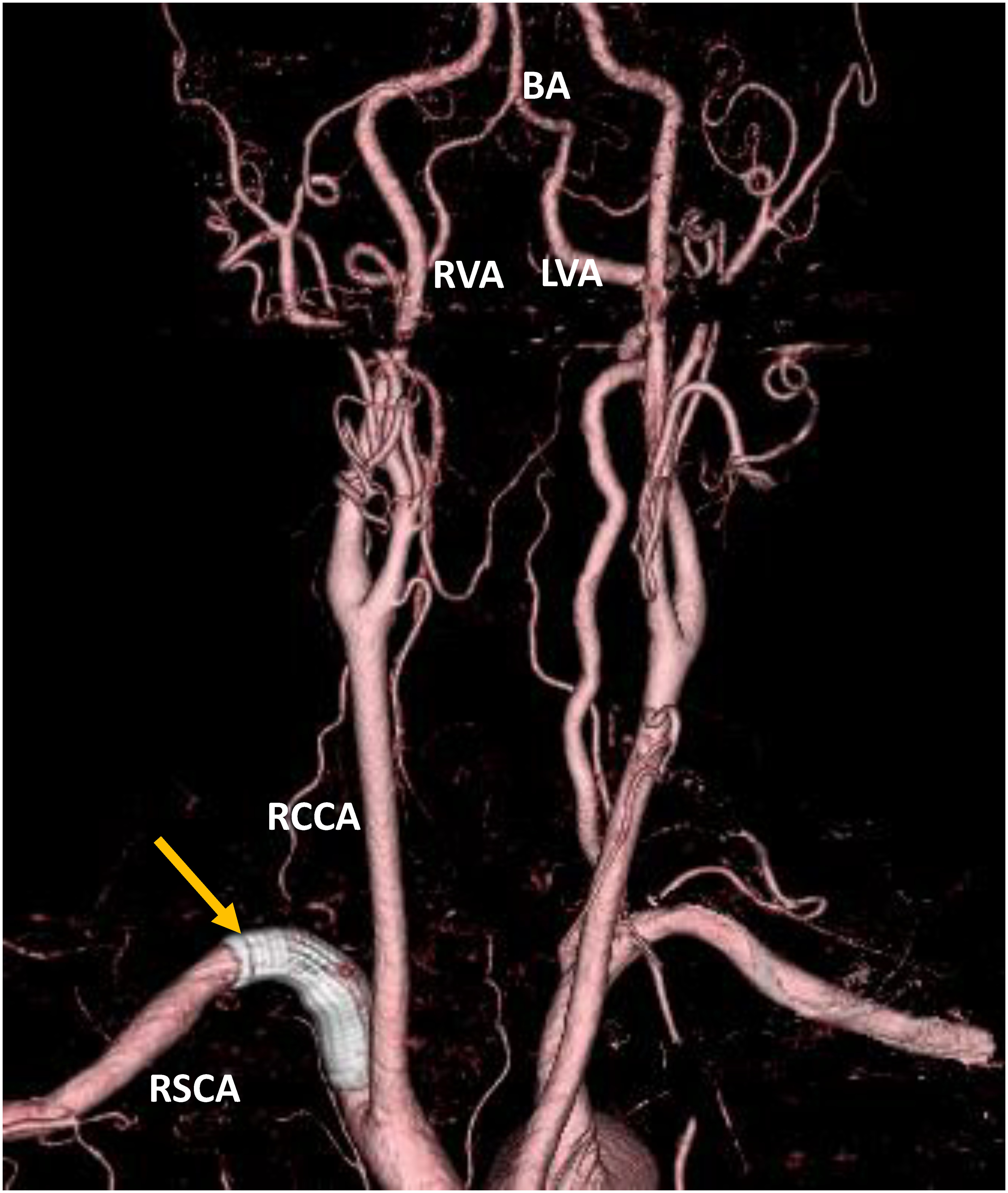
Fig. 3 Postoperative computed tomography. The arrow presented the covered stent.

BA: basilar artery; LVA: left vertebral artery; RCCA: right common carotid artery; RSCA: right subclavian artery; RVA: right vertebral artery

A 64-year-old man underwent aortic valve replacement with mitral valve repair 2 years earlier. At this procedure, the right subclavian artery was accidentally punctured during the placement of the central venous catheter through the right internal jugular vein, although he had exhibited no symptoms immediately after surgery. However, 2 years later, he presented weakness and numbness on the lateral side of his right arm and underwent a medical examination, which then revealed a bruit and thrill on his right neck. An enhanced computed tomography and angiography showed the AVF between the right subclavian artery and vertebral vein. The fistula site on the right subclavian artery was located contiguous with the vertebral and internal mammary arteries at 25 mm distal from the bifurcation of the brachiocephalic artery (**Figs. 1A** and **1B**). The vertebral vein and the epidural venous plexus were observed to be dilated (**Figs. 1B** and **1C**), which compressed the dura mater at levels of C1–5 (**Fig. 1D**). As the AVF causes reflux and dilatation of the perimedullary vein that resulted in congestive myelopathy with compressive symptoms, an interventional treatment was required. As it was predicted that pressurized and dilated veins complicated deep dissection around the fistula site, endovascular repair using a covered stent was considered as a first-line treatment. Although the placement of the covered stent would occlude the right vertebral artery, the intracranial basilar artery was fed by the bilateral vertebral arteries on preoperative computed tomography. Through a right subclavian incision, an 8-Fr sheath was introduced through the right axillary artery. A diagnostic angiography identified the fistula site on the right subclavian artery (**Fig. 2A**), and the elimination of the bruit and thrill on the neck was confirmed via a test occlusion of the proximal segment of the right subclavian artery with a 14×30 mm balloon. As per his intravascular ultrasound examination, it was revealed that the diameters of the right subclavian artery were 11.0 and 8.5 mm at the proximal and distal landing sites, respectively. After exchanging an 8-Fr sheath to a 12-Fr sheath, a 13×50 mm Viabahn stent graft (W. L. Gore, Flagstaff, AZ, USA) was deployed distally to the ostium of the right carotid artery to cover and occlude the fistula, followed by post-deployment angioplasty using a 14×30 mm balloon. The angiography showed a successful occlusion of the fistula, as well as the right vertebral and internal mammary arteries (**Fig. 2B**). His complaints were noted to disappear immediately after the operation. The postoperative course was uneventful. Computed tomography performed 1 week after the operation showed the patent covered stent, the basilar artery that was connected to the left vertebral artery, and no dilatation of the epidural veins (**Fig. 3**).

## Discussion

Mechanical complications caused by the placement of a central venous catheter in the internal jugular vein include arterial puncture (6.3%–9.4%), hematoma (<0.1%–2.2%), and pneumothorax (<0.1%–0.2%).^1)^ The consequences of arterial puncture are acute or delayed atherosclerotic thromboembolism, pseudoaneurysms, dissections, and AVF.^2)^ AVF is known to develop most frequently between the carotid artery and the jugular vein,^3)^ while axillo-subclavian and vertebro-vertebral AVF have also been reported.^4–6)^ In contrast, the AVF between the subclavian artery and the vertebral vein rarely develops. Although this AVF can be treated with open surgery or endovascular repair, there are several concerns that may affect the selection of treatment options. Open surgical repair for the injured subclavian artery near the vertebral artery is challenging. The major concern would be the depth of the treatment target that is associated with a difficulty in exposure and manipulations of the relevant vessels for repair. Expanded veins would also interfere with the procedure and may cause accidental injuries to vessels or nerves. In some cases, a sternotomy or thoracotomy would then be required. A report of open repair for axillo-subclavian arterial injury showed high mortality.^7)^ On the other hand, endovascular repair of the proximal segment of the right subclavian artery was associated with the risk of stroke and ischemia because there are several arteries branched, which could be occluded by the treatment.

However, because of less invasiveness, endovascular repair has been commonly selected as a first-line treatment if deemed anatomically feasible. Otherwise, open surgery should be indicated as well as in the case of treatment failure. A recent review on the treatment of vertebro-vertebral AVF showed that endovascular repair was selected in 73%–91%; the improvement rate of symptoms was 90%–97%; the rates of overall permanent morbidity and mortality related to treatment were 3.3% and 1.5%, respectively.^6)^ Although there are several treatment options including embolization with liquid embolic agents or coils, detachable balloons, and covered stents,^4–8)^ the reports that advocate the effectiveness of covered stent placement for the fistula involving the vertebral artery remained scarce possibly because its feasibility would be affected by the vessel diameter and the risk of branch occlusion.^6,9)^ In this present case, the right subclavian artery proximal to the fistula site was appropriate with respect to the length of the proximal landing zone and the diameter for the available Viabahn sizes. Although the occlusion of the right vertebral artery was inevitable, no cerebral infarction was noted to develop. As several reports showed that occlusion or ligation of one vertebral artery could be performed without any problem when the bilateral vertebral arteries were connected through the basilar arteries,^10)^ we confirmed the connection of the bilateral vertebral arteries at the vertebrobasilar junction on the preoperative computed tomography. In contrast, for the patients with a high predictability of postoperative stroke due to the occlusion of the vertebral artery, the chimney technique to maintain the patency of the artery should thus be considered. However, in this presented case, this technique may result in gutter endoleak, as the fistula site was very close to the ostium of the vertebral artery. In the endovascular repair, potentially suboptimal outcomes caused by endoleaks and other undetected fistula sites should be considered. In this present case, the intraoperative test occlusion using a balloon catheter helped judge the effectiveness of the covered stent placement.

Endovascular repair should be the first-line treatment for the fistula, especially if the fistula developed in the deep lesion where open repair is difficult to be adopted. However, in such lesions, there would be several important branch arteries. If occlusion of these branches is inevitable and is associated with a high risk of ischemic complications, endovascular repair should not be selected.

## Conclusion

The AVF between the right subclavian artery and the vertebral vein after central venous catheter placement is rare; moreover, it is difficult to treat with an open repair. Thus, endovascular repair with a covered stent should be first-line treatment if deemed anatomically feasible.
